# Nitric oxide synthase inhibition results in synergistic anti-tumour activity with melphalan and tumour necrosis factor alpha-based isolated limb perfusions

**DOI:** 10.1054/bjoc.2000.1447

**Published:** 2000-11

**Authors:** J H W de Wilt, E R Manusama, B van Etten, S T van Tiel, A S Jorna, A L B Seynhaeve, T L M ten Hagen

**Affiliations:** Department of Surgical Oncology, University Hospital Rotterdam/Daniel den Hoed Cancer Centre, Groene Hilledijk 301, Rotterdam, EA, 3075; Department of Pediatrics, Erasmus University Rotterdam, Rotterdam, The Netherlands

**Keywords:** L-NAME, melphalan, TNF, rats, perfusion

## Abstract

Nitric oxide (NO) is an important molecule in regulating tumour blood flow and stimulating tumour angiogenesis. Inhibition of NO synthase by L-NAME might induce an anti-tumour effect by limiting nutrients and oxygen to reach tumour tissue or affecting vascular growth. The anti-tumour effect of L-NAME after systemic administration was studied in a renal subcapsular CC531 adenocarcinoma model in rats. Moreover, regional administration of L-NAME, in combination with TNF and melphalan, was studied in an isolated limb perfusion (ILP) model using BN175 soft-tissue sarcomas. Systemic treatment with L-NAME inhibited growth of adenocarcinoma significantly but was accompanied by impaired renal function. In ILP, reduced tumour growth was observed when L-NAME was used alone. In combination with TNF or melphalan, L-NAME increased response rates significantly compared to perfusions without L-NAME (0–64% and 0–63% respectively). An additional anti-tumour effect was demonstrated when L-NAME was added to the synergistic combination of melphalan and TNF (responses increased from 70 to 100%). Inhibition of NO synthase reduces tumour growth both after systemic and regional (ILP) treatment. A synergistic anti-tumour effect of L-NAME is observed in combination with melphalan and/or TNF using ILP. These results indicate a possible role of L-NAME for the treatment of solid tumours in a systemic or regional setting. © 2000 Cancer Research Campaign


*British Journal of Cancer* (2000) **83**(9), 1176–1182
© 2000 Cancer Research Campaign
doi: 10.1054/ bjoc.2000.1447, available online at http://www.idealibrary.com on IDEAL®

### Nitric oxide synthase inhibition results in synergistic anti-tumour activity with melphalan and tumour necrosis factor alpha-based isolated limb perfusions

**JHW de Wilt[1], ER Manusama[1], B van Etten[1], ST van Tiel[1], AS Jorna[2], ALB Seynhaeve[1], TLM ten Hagen[1] and AMM Eggermont[1]**

[1]Department of Surgical Oncology, University Hospital Rotterdam/Daniel den Hoed Cancer Centre, Groene Hilledijk 301, 3075 EA Rotterdam; [2]Department of Pediatrics, Erasmus University Rotterdam, Rotterdam, The Netherlands

**Summary** Nitric oxide (NO) is an important molecule in regulating tumour blood flow and stimulating tumour angiogenesis. Inhibition of NO synthase by L-NAME might induce an anti-tumour effect by limiting nutrients and oxygen to reach tumour tissue or affecting vascular growth. The anti-tumour effect of L-NAME after systemic administration was studied in a renal subcapsular CC531 adenocarcinoma model in rats. Moreover, regional administration of L-NAME, in combination with TNF and melphalan, was studied in an isolated limb perfusion (ILP) model using BN175 soft-tissue sarcomas. Systemic treatment with L-NAME inhibited growth of adenocarcinoma significantly but was accompanied by impaired renal function. In ILP, reduced tumour growth was observed when L-NAME was used alone. In combination with TNF or melphalan, L-NAME increased response rates significantly compared to perfusions without L-NAME (0–64% and 0–63% respectively). An additional anti-tumour effect was demonstrated when L-NAME was added to the synergistic combination of melphalan and TNF (responses increased from 70 to 100%). Inhibition of NO synthase reduces tumour growth both after systemic and regional (ILP) treatment. A synergistic anti-tumour effect of L-NAME is observed in combination with melphalan and/or TNF using ILP. These results indicate a possible role of L-NAME for the treatment of solid tumours in a systemic or regional setting. © 2000 Cancer Research Campaign

**Keywords**: L-NAME; melphalan; TNF; rats; perfusion

Nitric oxide (NO) is a multi-functional messenger molecule derived from the amino acid, L-arginine, in a reaction catalysed by NO synthase (NOS). There are three isoforms of NOS: the calcium-dependent endothelial (eNOS) and neuronal (nNOS) and the calcium-independent inducible (iNOS = NOS2). High levels of NOS activity are present in several tumour cell lines as well as in human cancer (Thomsen et al, 1994; 1995). An important function of NO is to maintain or increase tumour blood flow via dilatation of arteriolar vessels in some tumours (Fukumura et al, 1997). This effect on tumour vasculature by NO enables vital nutrients and oxygen to reach tumour cells and can result in a promoted tumour growth in tumour cells that constantly release NO (Jenkins et al, 1995). Moreover, recent studies demonstrated another important effect of NO in stimulating tumour angiogenesis (Fukumura and Jain, 1998; Gallo et al, 1998; Thomsen and Miles 1998). Inhibition of NO synthase might inhibit tumour neovascularization and in this way reduce tumour growth.

Several authors demonstrated a selectively reduced tumour blood flow in rodents treated orally with NOS inhibitors, such as L-NAME (Andrade et al, 1992; Meyer et al; 1995; Tozer et al, 1998). Orucevic and Lala (1996*a*) demonstrated a concentration-dependent anti-tumour effect of L-NAME in adenocarcinoma-bearing mice. With other NO-inhibitors used as a single agent anti-tumour effects have been demonstrated as well (Thomsen et al, 1997). The reduction in tumour blood flow leads to hypoxia in tumour tissue and might thus be a useful strategy in anti-tumour therapy in combination with other agents. For alkylating agents such as melphalan and for cytokines such as tumour necrosis factor alpha (TNF) it has been demonstrated that hypoxia can potentiate the cytotoxic effects (Skarsgard et al, 1995; de Wilt et al, 1999).

To study the potential anti-tumour effects of systemic administration of L-NAME we used a renal subcapsular tumour model, using a colon carcinoma in WAG/Rij rats. Secondly, we examined whether addition of L-NAME to melphalan and/or TNF in an isolated limb perfusion (ILP) model could further improve response rates. For this we used a well-established perfusion model developed in our laboratory, which is based on the successful treatment of patients with in-transit metastasis from malignant melanoma (Liénard et al, 1992; Lejeune et al, 1993) and irresectable or locally advanced soft-tissue sarcoma (Eggermont et al, 1996*a*; 1996*b*). For the last group of patients TNF has recently been approved by the EMEA (European Medicine Evaluation Agency) in the ILP setting in combination with melphalan (Eggermont et al, 1999). In our ILP model strong synergistic anti-tumour effects were previously demonstrated when TNF was used in combination with two different chemotherapeutics (melphalan or doxorubicin) (Manusama et al, 1996; de Wilt et al, 1999; van der Veen et al, 2000). The synergistic anti-tumour effects were accompanied by higher intratumoural melphalan concentrations after perfusion with TNF compared to perfusions with melphalan alone (de Wilt et al, 2000). The observed effects in rats corresponded well to ILP in patients in terms of response rate and histopathological observations

*Received 3 February 2000*
*Revised 26 June 2000*
*Accepted 28 June 2000*

*Correspondence to:* AMM Eggermont

(Manusama et al, 1996; Nooijen et al, 1996). Therefore, this rat model is applied to study usefulness of additional agents in ILP to improve response rates or find synergy between agents, which allow lower dosages of toxic agents like TNF.

### MATERIALS AND METHODS

### Animals

Male inbred BN and Wag/Rij rats, weighing 250–300 g, obtained from Harlan-CPB (Austerlitz, the Netherlands) were used. The rats were fed a standard laboratory diet ad libitum (Hope Farms, Woerden, the Netherlands) and were housed under standard conditions. The experimental protocols adhered to the rules outlined in the Dutch Animal Experimentation Act (1977) and the published 'Guidelines on the protection of Experimental Animals' by the council of the European Committee (1986). The protocol was approved by the committee on Animal Research of the Erasmus University Rotterdam, The Netherlands.

### Drugs

Melphalan (Alkeran, 50 mg per vial, Wellcome, Beckenham, UK) was diluted in 10 ml diluent solvent. Further dilutions were made in 0.9% NaCl to give a volume of 0.2 ml in the perfusion circuit (= 40 µg). Recombinant human TNF alpha (TNF) was provided by Boehringer (Ingelheim, Germany) having a specific activity of $5.8 \times 10^7$ U $mg^{-1}$ as determined in the murine L-M cell assay (Kramer and Carver, 1986). Endotoxin levels were < 1.25 endotoxin units (EU) per mg protein. $N_\omega$-nitro-L-arginine methyl ester (L-NAME) (10 g per vial, Sigma, the Netherlands) was dissolved in 0.9% NaCl and administered intraperitoneally at a concentration of 80 mg $kg^{-1}$ or dissolved in Haemaccel and added to the perfusate to provide a concentration of 2 mg $ml^{-1}$.

### Western blot analysis for detection of inducible nitric oxide synthesis (iNOS)

Protein extracts were prepared from tissue pieces crushed under liquid nitrogen and homogenized on ice in RIPA buffer (50 mM Tris-HCl, 150 mM NaCl, 1% Triton X-100, 1% deoxycholate, and 0.1% SDS) containing 1 mM DTT, 0.1 mM PMSF and 10 mg $l^{-1}$ aprotinin. Supernatant was prepared by centrifugation at 120 000 ***g*** for 10 min, and protein concentrations were determined with the Coomassie Plus Protein Reagent (Pierce IL, USA). One-dimensional Western blot analysis was carried out, for detection of iNOS (Towbin et al, 1979). Briefly, sodium dodecyl sulphate polyacrylamide gel electrophoresis (SDS-PAGE) was carried out in the Biorad minigel system with 7% polyacrylamide gel using 300 µg of soluble protein extracts. Electrophoresed proteins were transfered to a PVDF membrane (Millipore Corp, MA, USA) and unspecific binding was blocked by incubation of the membrane in TBST (10 mM Tris, 150 mM NaCl, and 0.05% Tween 20) plus 2% BSA for 1 h at room temperature. The membranes were probed with a polyclonal rabbit anti-rat iNOS antibody (N-20, Santa Cruz Biotechnology Inc, CA, USA), diluted 1:40 000 in TBST. iNOS antibody was detected using a secondary mouse antibody to rabbit which was alkaline phosphatase labelled (Sigma, St. Louis MO, USA). Colour development was performed using the alkaline phosphate substrate nitroblue tetrazolium (NBT) and 5-bromo-4-chloro-3-indolyl-phosphate (BCIP) in AF-buffer until colour was fully developed (Boehringer Mannheim, Mannheim, Germany).

### Immunohistochemical staining of tumour samples for iNOS.

Portions of BN-175 tumour samples embedded in paraffin and 4µm section were collected on clean glass slides. Slides were incubated with rabbit polyclonal anti iNOS (NOS2) antibody (N20, Santa Cruz), followed by incubation with a secondary goat-anti-rabbit peroxidase conjugated antibody (Jackson Immuno-Research Laboratories Inc, West Grove PA, USA). Colour was developed using DAB reagent according to the manufacturer (Sigma).

### Renal sub-capsular tumour model

Renal sub-capsular tumour model was established in male rats of the inbred WAG-Rij strain introducing 8 mg of solid CC531 colon carcinoma under the capsule of both kidneys under microscopic vision, according to a previously described method (Marquet et al, 1984). Treatment was started 1 day after implantation by intraperitoneal injection of 80 mg $kg^{-1}$ L-NAME twice daily. Control rats were treated with a phosphate-buffered saline (PBS) solution. After 10 days of treatment rats were sacrificed and kidney tumours weighed. Both groups consisted of eight rats and all animals were evaluable. With respect to systemic toxicity of L-NAME, body weights of the rats were measured 4 and 10 days after treatment and creatinine and urea levels were determined at sacrifice.

### Isolated limb perfusion model

The technique we used has been published previously (Manusama et al, 1996). Briefly, a spontaneous, non-immunogenic BN-175 sarcoma was used and implanted subcutaneously in the right hind limb in BN rats (Kort et al, 1984). Perfusion was performed at a tumour diameter of 13 ± 3 mm at least 7 days after implantation. Animals were anaesthetized with Hypnorm (Janssen Pharmaceutica, Tilburg, The Netherlands) and 50 IU of heparin were injected intravenously to prevent coagulation in the perfusion circuit. A warm water mattress was applied to maintain a constant temperature of 38–39°C in the hind limb during perfusion. The femoral artery and vein were cannulated with silastic tubing 0.30 mm ID, 0.64 mm OD; 0.64 mm ID 1.15 mm OD respectively, Dow Corning, Michigan, USA). Collaterals were occluded by a groin tourniquet and isolation time started when the tourniquet was tightened. An oxygenation reservoir and a roller pump were included into the circuit. The perfusion commenced with 5 ml Haemaccel (Behring Pharma, Amsterdam, the Netherlands) and the haemoglobin (Hb) content of the perfusate was 0.9 mmol $l^{-1}$. L-NAME was dissolved in the perfusate, melphalan and TNF were added as boluses to the oxygenation reservoir. A roller pump (type 505 U; Watson Marlow, Falmouth, UK) recirculated the perfusate at a flow rate of 2.4 ml $min^{-1}$. A washout with 2 ml oxygenated Haemaccel was performed at the end of the perfusion. In the rat collateral circulation via the internal iliac artery to the leg is so extensive that it allows ligation of the femoral vessels without detrimental effects. After ligation of the femoral artery back-flow from the femoral vein was seen in all rats immediately after release of the tourniquet.

*© 2000 Cancer Research Campaign* 

Subsequent tumour growth was daily recorded by calliper measurement. Tumour volume was calculated as 0.4 ($A^2B$), where B represents the longest diameter and A the diameter perpendicular to B.

### Assessment of tumour response

The classification of tumour response was: progressive disease (PD) = increase of tumour volume (> 25%) within 4 days; no change (NC) = tumour volume equal to volume during perfusion (in a range of –25% – +25%); partial remission (PR) = decrease of tumour volume (–25 – –90%); complete remission (CR) = tumour volume 0–10% of volume during perfusion or necrosis.

### Statistical analysis

Mann–Whitney U test was used to compare tumour volumes in different animal groups and to compare different tumour responses in different groups. Calculations were performed on a personal computer using GraphPad Prism and SPSS for Windows 95.

### RESULTS

### iNOS Western blot

Western blot analysis of tumor extracts demonstrated distinct iNOS bands at approximately $M_r$ 125 000–138 000 (Ambs et al, 1998). iNOS was demonstrated in tumour tissue but not in muscle tissue (Figure 1). The results suggest that iNOS is more abundant in tumour tissue than in normal muscle tissue in the rat and suggest an important role for iNOS in tumour tissue.

### iNOS immunohistochemistry

The source of intratumoural NO production was determined on BN-175 tumour slides by immunohistochemistry with a polyclonal anti-NOS2 antibodies. In Figure 2 clear production of iNOS in tumour cells is shown. These results correspond with in vitro observations showing NO production by BN-175 tumour cells after stimulation with mitogens.

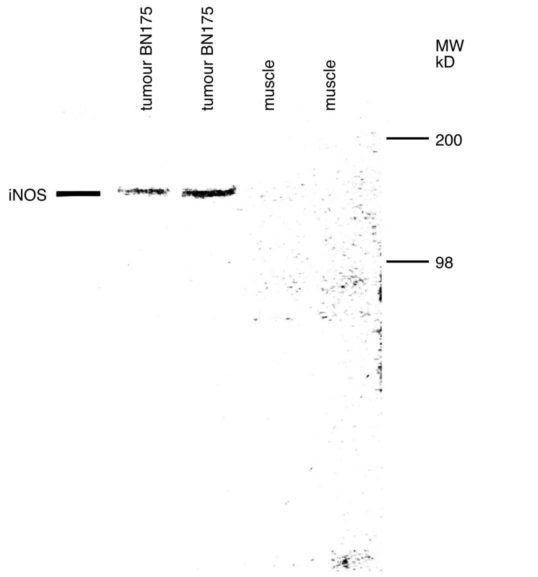

**Figure 1** Western blot analysis showing a distinct band at approximately 125–138 kD

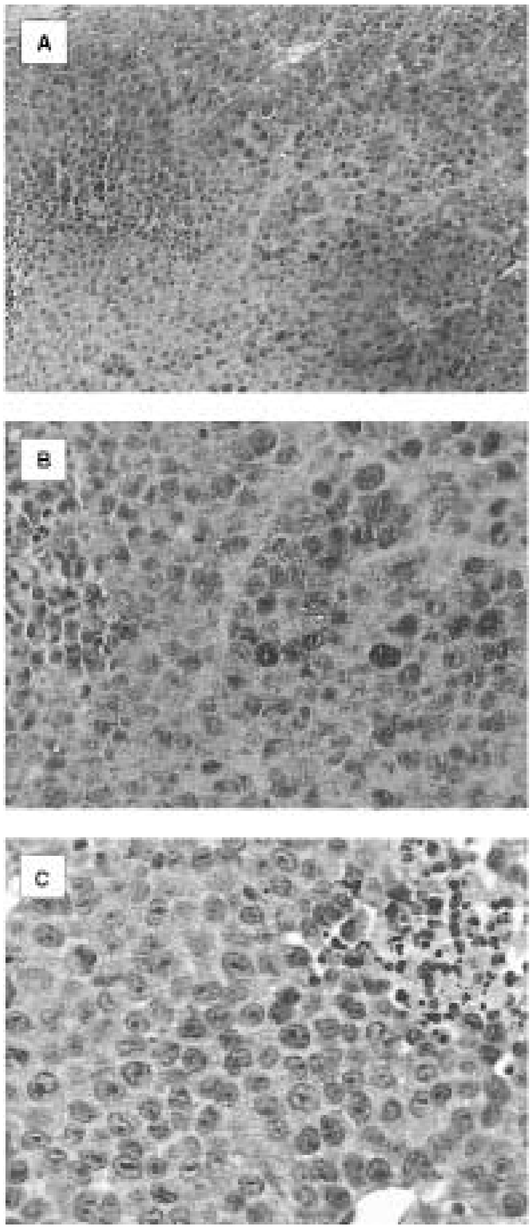

**Figure 2** Paraffin tissue sections from soft-tissue sarcoma BN-175 stained with anti-NOS2 (iNOS). Nitric oxide synthesis-positive tumour cells (brown staining) can be found throughout the section as shown in (**A**) and (**B**) (respectively original magnification of × 16 and × 40), whereas infiltrating cells stain negative. No staining was found in negative control slides in which non-specific IgG was used (**C**, original magnification × 40).

### Renal sub-capsular tumour model

Systemic treatment with L-NAME resulted in a statistically significant growth inhibition of CC-531 colon carcinoma growing under the kidney capsule compared to untreated rats (44.8 vs 64.9 g; $P < 0.005$) (Fig. 3). Ten days after treatment, body weight of rats were statistically not significantly different between both groups (–13.0 ± 5.4 g after L-NAME treatment vs –5.5 ± 3.1 g after sham

 *© 2000 Cancer Research Campaign*

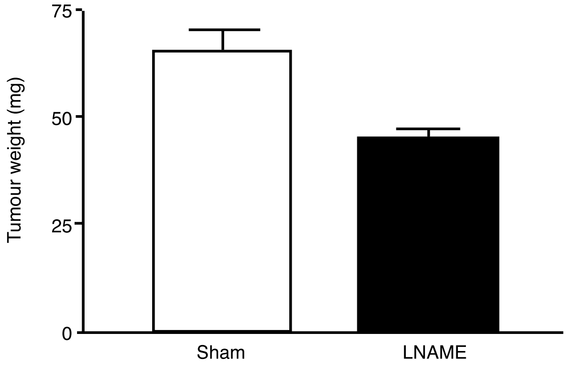

**Figure 3** Tumour weight (mean +/– SEM) of renal subcapsular CC531 adenocarcinoma after 10 days treatment with sham (*n* = 8) or 80 mg kg$^{-1}$ L-NAME intraperitoneal injection (*n* = 8)

treatment). At day 10 after intraperitoneal administration creatinine and urea levels were statistically significantly different from the control group (creatinine 28.5 ± 4.3 vs 68.5 ± 8.5 μmol l$^{-1}$ ($P < 0.005$) and urea 5.6 ± 0.4 vs 8.1 ± 1.3 mmol l$^{-1}$ ($P < 0.05$) in the control and L-NAME groups respectively). This increase in urea and creatinine levels indicate a decrease in renal function that might be the result of impaired renal blood flow.

### Tumour response after isolated limb perfusions with L-NAME, TNF and/or melphalan

We studied the possible beneficial role of L-NAME on tumour response in ILP. Synergy between melphalan and TNF in ILP was previously demonstrated in our laboratory and could be confirmed in this study for which we used 10 rats in each study group (Table 1) (de Wilt et al, 1999; Manusama et al, 1996). Sham perfusion did not inhibit tumour growth and progressive disease was observed in all rats. Perfusions with L-NAME as a single agent, however, resulted in tumour growth arrest after 5 days in four of 11 rats, which was statistically significant different from sham ILP ($P = 0.02$), resulting in a growth delay as shown in Figure 4A.

ILP with TNF alone resulted in progressive disease in all animals, similar as in sham perfused rats. Addition of L-NAME to TNF improved tumour responses from 0–64%, which was significantly different from TNF alone ($P < 0.001$).

After perfusion with melphalan tumour growth was arrested in eight of 10 animals (no change) and progressive tumour growth was observed in two of 10 animals. Melphalan in combination with L-NAME showed a 63% partial and complete response rate, which was significantly different from melphalan alone ($P$ = 0.001).

TNF and melphalan have a synergistic anti-tumour effect and are highly effective with a 70% partial and complete response rate. Addition of L-NAME to the combination of TNF and melphalan, however, could further improve tumour responses to 100% 5 days after treatment, but this was not statistically

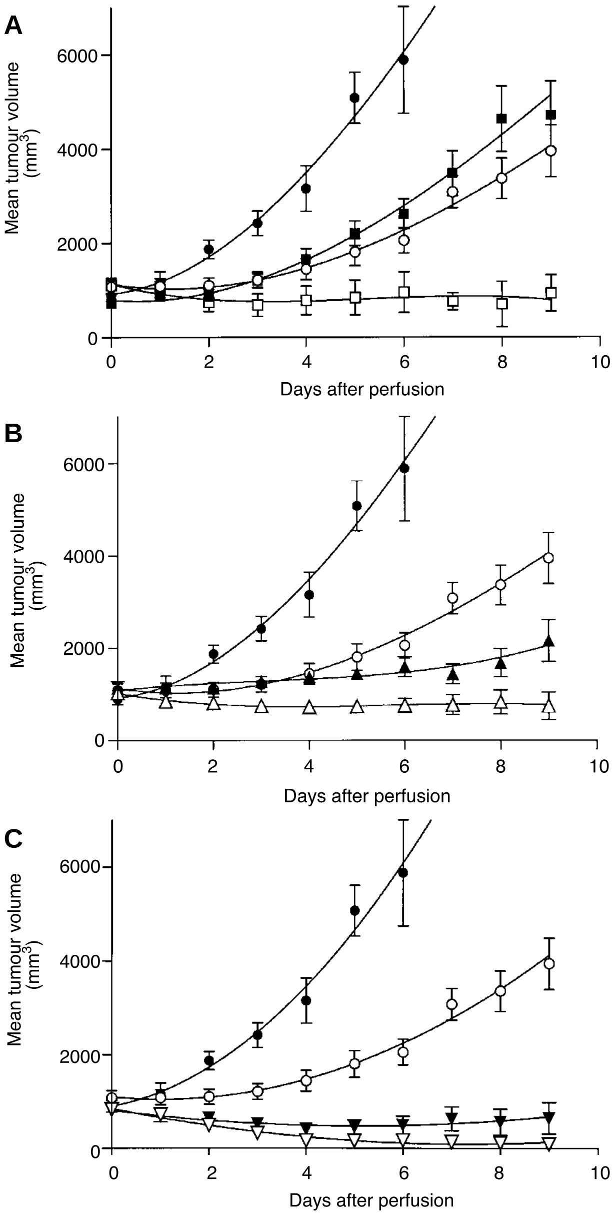

**Figure 4** (**A**, **B** and **C**) Growth curves of BN175 sarcoma after isolated limb perfusion with sham (●; *n* = 10), 10 mg L-NAME (○; *n* = 11), 50 μg TNF (■; *n* = 10), 50 μg TNF with 10 mg L-NAME (□; *n* = 11), 40 μg melphalan (▲; *n* = 10), 40 μg melphalan with 10 mg L-NAME (△; *n* = 16), 50 μg TNF in combination with 40 μg melphalan (▼; *n* = 10) and 50 μg TNF in combination with 40 μg melphalan and 10 mg L-NAME (▽) *n* = 10). Growth curves of sham and L-NAME perfused rats is depicted in all figures to allow comparison (mean +/– SEM of tumour volumes are shown)

significant ($P$ = 0.3) After perfusion with TNF and melphalan recurrent tumour growth was demonstrated in all animals after a mean of 9 ± 2 days. When L-NAME was added to the perfusate one animal did not show tumour growth 50 days after ILP, whereas recurrent tumour growth occurred in nine of 10 animals after a mean of 20 ± 7 days (data not shown). The observed response rate was therefore not only more pronounced but the anti-tumour effect extended for a significantly longer period after perfusion.

© 2000 Cancer Research Campaign

**Table 1** Tumour response of BN-175 soft-tissue sarcoma after isolated limb perfusion

| Tumour response | Sham (*n* = 10) | Sham + LNAME (*n* = 11) | TNF (*n* = 10) | TNF + LNAME (*n* = 11) | Melphalan (*n* = 10) | Mel + LNAME (*n* = 16) | TNF + Melphalan (*n* = 10) | TNF + Mel + LNAME (*n* = 10) |
|---|---|---|---|---|---|---|---|---|
| Progressive disease | 10 | 7 | 10 | 3 | 2 | 1 | 1 | – |
| No change | – | 4 | – | 1 | 8 | 5 | 3 | – |
| Partial response | – | – | – | 6 | – | 5 | 1 | 3 |
| Complete response | – | – | – | 1 | – | 5 | 6 | 7 |
| Percentage response (partial/complete) | – | – | – | 64% | – | 63% | 70% | 100% |

Perfusions were performed with 50 μg TNF, 40 μg melphalan and 10 mg L-NAME under constant temperature (38–39°C) for 30 min

### DISCUSSION

The results of the present study show a significantly reduced tumour growth after intraperitoneal administration of the nitric oxide (NO) inhibitor L-NAME in tumour-bearing rats. The growth inhibition of L-NAME in the renal sub-capsular assay may be partially due to a decrease in renal blood flow. Kassab et al (1998) previously demonstrated a decreased renal blood flow after administration of NO inhibitors, which is confirmed in our study by elevated creatinine and urea levels. Therefore, conclusions concerning anti-tumour effect of NO inhibitors from the data obtained in the renal sub-capsular assay cannot be drawn, but these data strongly suggest a growth inhibitory effect on the tumour by NO inhibitors.

In the experiments in which L-NAME was used in an isolated perfusion setting in sarcoma-bearing rats a decreased tumour growth was demonstrated when L-NAME was used alone. Moreover, strong synergy was observed when L-NAME was used in combination with either TNF (response rates improved from 0–64%) or melphalan (response rates improved from 0–63%). Even in the setting of the strongly synergistic combination of melphalan and TNF (response rates: 70%) the addition of L-NAME enhanced response rates to 100%. Moreover, when L-NAME was added to the perfusion tumour growth recurrences occurred at approximately 20 days after ILP, whereas tumours recur after a mean of 9 days after ILP with TNF and melphalan alone.

The anti-tumour effects of L-NAME in the highly vascularized BN-175 soft tissue sarcoma found in this study are similar to previously demonstrated effects of NO inhibitors in mice (Orucivic and Lala, 1996a; Thomsen et al, 1997). The function of NO inhibition in tumour biology, however, is not clear since NO has a multifactorial role in the vascular, nervous and immune system and is demonstrated in many different cell lines and tissues. High concentrations of NO synthase (NOS) are present in different tumour cell lines, where the enzyme activity correlates with the tumour grade (Thomsen et al, 1995; Ambs et al, 1998). We demonstrated iNOS to be present in the BN-175 soft-tissue sarcoma and not in the surrounding muscle tissue using a Western blott analysis and immunohistochemistry. High levels of NO induced by iNOS suppresses metastatic potential and directly correlates with cytotoxicity in several studies (Xie and Fidler, 1999). In contrast, Jenkins et al (1995) found a promotion of tumour growth in tumour cells that constantly produce NO. Recently others demonstrated that iNOS activity was higher in metastasizing head and neck cancer tissue compared to normal tissue, suggesting an important role for NO in tumour biology (Gallo et al, 1998).

One mechanism of NO is to increase or maintain tumour blood flow and therefore supply nutrients and oxygen to the tumour (Buttery et al, 1993). In studies in which rodents were treated with NO inhibitors, a selectively reduced tumour blood flow was demonstrated (Andrade et al, 1992; Tozer et al, 1997). This decreased flow is initiated by a decreased central tumour perfusion in some tumours (Meyer et al, 1995) or a decreased peripheral perfusion in others (Fukumura et al, 1997). Localization of NOS is cell- and tumour-dependent and as a result the response to NO inhibitor is likely to be heterogeneous and tumour-dependent. Horsman et al (1996) did not find a decrease in oxygenation status of tumours treated with NO inhibitors despite a significantly reduced tumour blood flow. However, Wood et al (1994) demonstrated that reduced flow by an NO inhibitor decreased the energy status of several murine tumours, whereas the normal skin was unaffected. Moreover, they found evidence for an increase in tumour sensitivity to a level sufficient to enhance the efficacy of cytostatic agents (Wood et al, 1994). In vivo studies have shown that reduction of tumour blood flow with agents such as hydralazine can enhance the tumouricidal effect of melphalan. In vitro studies on human tumour cells also demonstrated a potentiation of melphalan cytotoxicity by both hypoxia and acidic pH (Skarsgard et al, 1995). We previously demonstrated promotion of TNF as well as melphalan anti-tumour effects with hypoxia in soft-tissue sarcoma-bearing rats in ILP (de Wilt et al, 1991). The enhanced anti-tumour effect of L-NAME with melphalan and/or TNF as we describe in this study might thus be explained by hypoxia that is induced by the reduced tumour blood flow.

More recently an important role of NO in tumour angiogenesis was suggested (Fukumura and Jain, 1998; Thomsen and Miles, 1998). NO was shown to promote tumour progression by down-regulating tissue inhibitors of metalloproteinases (TIMP) and up-regulating matrix metalloproteinases (MMP) (Orucevic et al, 1999). Gallo et al (1998) demonstrated that inhibition of NO by L-NAME in squamous cell carcinoma transplanted in the rabbit cornea decreased tumour-induced angiogensis. Similar reduced tumour angiogenesis by L-NAME was demonstrated in a highly metastatic murine breast cancer model by Jadeski and Lala (1999). Since BN-175 is a highly vascularized and fast-growing tumour, inhibition of neovascularization in this tumour might well be a good explanation for the significant tumour responses and later re-growth after perfusion with L-NAME in combination with melphalan and TNF.

Leukocyte–endothelial interactions in tumour vessels is a major limitation of immune therapy or host immune response against tumours. NO has a possible role in down-regulating these actions and inhibition of NO was demonstrated to increase leukocyte rolling and adhesion to the vessel wall in tumours significantly (Fukumura et al, 1995). Lejeune et al (1994) found an enhanced tumour-infiltrating lymphocyte proliferation in rat colon adenocarcinoma when NO production was inhibited with

 *© 2000 Cancer Research Campaign*

L-NAME. Previous work in our laboratory and by others demonstrated that TNF-induced anti-tumour effect might be leukocyte-dependent (Renard et al, 1994; Fukumura et al, 1995; Manusama et al, 1998). Increased leukocyte–endothelial interactions induced by L-NAME might therefore be another reason for the additional anti-tumour effect of L-NAME to TNF. Orucevic and Lala (1996b) previously demonstrated an increased anti-tumour effect when L-NAME was combined with another cytokine (e.g. IL-2). Meyer et al (1995) suggested that the vascular effects they observed in tumours treated with L-NAME was not only due to leukocyte adhesion but also to the development of microthrombi resulting from platelet aggregation. Since platelet aggregation is an important event in the TNF anti-tumour response as well, this mechanism might be another reason for the enhanced effect of L-NAME with TNF (Renard et al, 1995; Nooijen et al, 1996).

In conclusion, we demonstrate that L-NAME leads to a reduction in tumour growth both after systemic administration as well as in ILP against a non-immunogenic soft-tissue sarcoma in the rat. The observed high response rates with the addition of L-NAME to melphalan and TNF are promising for the use of L-NAME in the clinical setting. Since the dose-limiting toxic effect of TNF in cancer therapy is mainly hypotension induced by NO production in endothelial cells, systemic L-NAME may favourably alter this toxicity which could result in a higher maximum tolerated dose (Kilbourn and Belloni, 1990; Kilbourn et al, 1990). This might open therapeutical options for TNF cancer treatment in other settings than in ILP. Optimization of NOS inhibitor concentrations and kinetics will be necessary to fully exploit this potential therapy.

### REFERENCES

Ambs S, Merriam WG, Bennett WP, Felley-Bosco E, Ogunfusika MO, Oser SM, Klein S, Shields PG, Billiar TR and Harris CC (1998) Frequent nitric oxide synthase-2 expression in human colon adenomas: implications for tumour angiogenesis and colon cancer progression. *Cancer Res* **58**: 334–341

Andrade SP, Hart IR and Piper PJ (1992) Inhibitors of nitric oxide synthase selectively reduce flow in tumour-associated neovasculature. *Br J Pharmacol* **107**: 1092–1095

Buttery LDK, Springall DR, Andrade SP, Riveros-Moreno V, Hart I, Piper PJ and Polak JM (1993) Induction of nitric oxide synthase in the neo-vasculature of experimental tumours in mice. *J Pathol* **171**: 311–319

de Wilt JHW, Manusama ER, van Tiel ST, van IJken MGA, ten Hagen TLM and Eggermont AMM (1999) Prerequisites for effective isolated limb perfusion using tumour necrosis factor alpha and melphalan in rats. *Br J Cancer* **80**: 161–166

de Wilt JHW, ten Hagen TLM, de Boeck G, van Tiel ST, de Bruijn EA and Eggermont AMM (2000) Tumour necrosis factor alpha increases melphalan concentration in tumour tissue after isolated limb perfusion. *Br J Cancer* **82**: 1000–1003

Eggermont AMM, Schraffordt Koops H, Liénard D, Kroon BBR, van Geel AN, Hoekstra HJ and Lejeune FJ (1996*a*) Isolated limb perfusion with high-dose tumor necrosis factor-α in combination with interferon-γ and melphalan for nonresectable extremity soft tissue sarcomas: a multicenter trial. *J Clin Oncol* **14**: 2653–2665

Eggermont AMM, Schraffordt Koops H, Klausner J, Kroon BBR, Schlag PM, Liénard D, van Geel AN, Hoekstra HJ, Meller I, Nieweg OE, Kettelhack C, Ben-Ari G, Pector JC and Lejeune FJ (1996*b*) Isolated limb perfusion with tumor necrosis factor and melphalan for limb salvage in 186 patients with locally advanced soft tissue extremity sarcomas: the cumulative multicenter european experience. *Ann Surg* **224**: 756–765

Eggermont AMM, Schraffordt Koops H, Klausner JM, Schlag PM, Lienard D, Kroon BBR, Gustafson P, Steinmann G, Clarke J and Lejeune FJ (1999) Limb salvage by isolated limb perfusion (ILP) with TNF and melphalan in patients with locally advanced soft tissue sarcomas: outcome of 270 ILPs in 246 patients. *Proc ASCO* **18**: 2067

Fukumura D and Jain RK (1998) Role of nitric oxide in angiogenesis and microciruculation in tumors. *Cancer Metastasis Rev* **17**: 77–89

Fukumura D, Salehi HA, Witwer B, Tuma RF, Melder RJ and Jain RK (1995) Tumour necrosis factor α-induced leukocyte adhesion in normal and tumour vessels: effect of tumour type, transplantation site, and host strain. *Cancer Res*: **55**: 4824–4829

Fukumura D, Yuan F, Endo M and Jain RK (1997) Role of nitric oxide in tumour microcirculation: blood flow, vascular permeability, and leukocyte – endothelial interactions. *Am J Pathol* **150**: 713–725

Gallo O, Masini E, Morbidelli L, Franchi A, Fini-Storchi I, Vergari WA and Ziche M (1998) Role of nitric oxide in angiogenesis and tumour progression in head and neck cancer. *J Natl Cancer Inst* **90**: 587–596

Horsman MR, Chaplin DJ, Hill SA, Arnold S, Collingridge D, Radacic M, Wood PJ and Overgaard J (1996) Effect of nitro-L-arginine on blood flow, oxygenation and the activity of hypoxic cell cytotoxins in murine tumours. *Br J Cancer* **74**: S168–171

Jadeski LC and Lala PK (1999) Nitric oxide synthase inhibition by N(G)-nitro-L-arginine methyl ester inhibits tumor-induced angiogenesis in mammary tumors. *Am J Pathol* **155**: 1381–1390.

Jenkins DC, Charles IG, Thomsen LL, Moss DW, Holmes LS, Baylis SA, Rhodes P, Westmore K, Emson PC and Moncada S (1995) Roles of nitric oxide in tumour growth. *Proc Natl Acad Sci* **92**: 4392–4396

Kassab S, Miller MT, Hester R, Novak J and Granger JP (1998) Systemic hemodynamics and regional blood flow during chronic nitric oxide synthesis inhibition in pregnant rats. *Hypertension* **31**: 315–320

Kilbourn RG and Belloni P (1990) Endothelial cell production of nitrogen oxides in response to interferon γ in combination with tumour necrosis factor, interleukin-1, or endotoxin. *J Natl Cancer Inst* **82**: 772–776

Kilbourn RG, Gross SS, Jubran A, Adams J, Griffith OW, Levi R and Lodato RF (1990) $N^G$-nitro-L-arginine inhibits tumour necrosis factor-induced hypotension: implications for the involvement of nitric oxide. *Proc Natl Acad Sci USA* **87**: 3629–3632

Kort WJ, Zondervan PE, Hulsman LO, Weijma IM and Westbroek DL (1984) Incidence of spontaneous tumors in a group of retired breeder female brown Norway rats. *J Natl Cancer Inst* **72**: 709–713

Kramer SM and Carver ME (1986) Serum-free in vitro bioassay for the detection of tumor necrosis factor. *J Immunol Methods* **93**: 201–206

Lejeune FJ, Liénard D, Leyvraz S and Mirimanoff RO (1993) Regional therapy of melanoma. *Eur J Cancer* **29A**: 606–612

Lejeune P, Lagadec P, Onier N, Pinard D, Ohshima H and Jeannin JF (1994) Nitric oxide involvement in tumour-induced immunosuppression. *J Immunol* 52: 5077–5083

Liénard D, Ewalenko P, Delmotte JJ, Renard N and Lejeune FJ (1992) High-dose recombinant tumor necrosis factor alpha in combination with interferon gamma and melphalan in isolation perfusion of the limbs for melanoma and sarcoma. *J Clin Oncol* **10**: 52–60

Manusama ER, Nooijen PTGA, Stavast J, Durante NMC, Marquet RL and Eggermont AMM (1996) Synergistic anti-tumour effect of recombinant human tumour necrosis factor α with melphalan in isolated limb perfusion in the rat. *Br J Surg* **83**: 551–555

Manusama ER, Nooijen PTGA, Stavast J, de Wilt JHW, Marquet RL and Eggermont AMM (1998) Assessment of the role of neutrophils on the antitumor effect of TNFα in an in vivo isolated limb perfusion model in sarcoma-bearing brown norway rats. *J Surg Res* **78**: 169–175

Marquet RL, Westbroek DL and Jeekel J (1984) Interferon treatment of a transplantable rat colon adenocarcinoma: importance of tumour site. *Int J Cancer* **33**: 689–92

Meyer RE, Shan S, DeAngelo J, Dodge RK, Bonaventura J, Ong ET and Dewhirst MW (1995) Nitric oxide synthase inhibition irreversibly decreases perfusion in the R3230Ac rat mammary adenocarcinoma. *Br J Cancer* **71**: 1169–1174

Nooijen PTGA, Manusama ER, Eggermont AMM, Schalkwijk L, Stavast J, Marquet RL, de Waal RMW and Ruiter DJ (1996) Synergistic anti-tumour effects of TNF-α and melphalan in an isolated limb perfusion model of rat sarcoma: a histopathologic, immunohistochemical and electron microscopic study. *Br J Cancer* **74**: 1908–1915

Orucivic A and Lala PK (1996*a*) $N^G$-nitro-L-arginine methyl ester, an inhibitor of nitric oxide synthesis, ameliorates interleukin 2-induced capillary leakage and reduces tumour growth in adenocarcinoma-bearing mice. *Br J Cancer* **73**: 189–196

Orucevic A and Lala PK (1996*b*) Effects of N(G)-Nitro-L-arginine methyl ester, an inhibitor of nitric oxide synthesis, on IL-2-induced LAK cell generation in vivo and in vitro in healthy and tumor-bearing mice. *Cell Immunol* **169**: 125–32

Orucevic A, Bechberger J, Green AM, Shapiro RA, Billiar TR and Lala PK (1999) Nitric-oxide production by murine mammary adenocarcinoma cells promotes tumor-cell invasiveness. *Int J Cancer* **81**: 889–896

*© 2000 Cancer Research Campaign*

Renard N, Liénard D, Lespagnard L, Eggermont A, Heimann R and Lejeune F (1994) Early endothelium activation and polymorphonuclear cell invasion precede specific necrosis of human melanoma and sarcoma treated by intravascular high-dose tumour necrosis factor alpha (rTNFα). *Int J Cancer* **57**: 656–663

Renard N, Nooijen PTGA, Schalkwijk L, de Waal RMW, Eggermont AMM, Liénard D, Kroon BBR, Lejeune FJ and Ruiter DJ (1995) VWF release and platelet aggregation in human melanoma after perfusion with TNFα. *J Pathol* **176**: 279–287

Skarsgard LD, Skwarchuk MW, Vinczan A, Kristl J and Chaplin DJ (1995) The cytotoxicity of melphalan and its relationship to pH, hypoxia and drug uptake. *Anticancer Res* **15**: 219–224

Thomsen LL and Miles DW (1998) Role of nitric oxide in tumour progression: lessons from human tumours. *Cancer Metastasis Rev* **17**: 107–118.

Thomsen LL, Lawton FG, Knowles RG, Beesley JE, Riveros-Moreno V and Moncada S (1994) Nitric oxide synthase activity in human gynecological cancer. *Cancer Res* **54**: 1352–1354

Thomsen LL, Miles DW, Happerfield L, Bobrow LG, Knowles RG and Moncada S (1995) Nitric oxide synthase activity in human breast cancer. *Br J Cancer* **72**: 41–44

Thomsen LL, Scott JMJ, Topley P, Knowles RG, Keerie A-J and Frend AJ (1997) Selective inhibition of inducible nitric oxide synthase inhibits tumour growth in vivo: studies with 1400W, a novel inhibitor. *Cancer Res* **57**: 3300–3304

Towbin H, Staehalin T and Gordon J (1979) Electrophoretic transfer of proteins from polyacrylamide gels to nitrocellulose sheets: procedure and some application. *Proc Natl Acad Sci USA* **76**: 4350–4354

Tozer GM, Prise VE and Chaplin DJ (1997) Inhibition of nitric oxide synthae induces a selective reduction in tumour blood flow that is reversible with L-arginine. *Cancer Res* **57**: 948–955

van der Veen AH, de Wilt JHW, Eggermont AMM, van Tiel ST, Seynhaeve ALB and ten Hagen TLM (2000) TNF augments intratumoural concentrations of doxorubicin in TNF-based isolated limb perfusion in rat sarcoma models and enhances antitumour effects. *Br J Cancer* **82**: 973–980

Wood PJ, Sansom JM, Butler SA, Stratford IJ, Cole SM, Szabo C, Thiemermann C and Adams GE (1994) Induction of hypoxia in experimental murine tumors by the nitric oxide synthase inhibitor, $N^G$-nitro-L-arginine. *Cancer Res* **54**: 6458–6463.

Xie K and Fidler IJ (1999) Therapy of cancer metastasis by activation of the inducible nitric oxide synthase. *Cancer Metastasis Rev* **17**: 55–75

 *© 2000 Cancer Research Campaign*
